# What determines mortality in malignant pheochromocytoma? – Report of a case with eighteen-year survival and review of the literature

**DOI:** 10.20945/2359-3997000000033

**Published:** 2018-03-23

**Authors:** Matheus de Oliveira Andrade, Vinícius Santos da Cunha, Dayana Carla de Oliveira, Olívia Laquis de Moraes, Adriana Lofrano-Porto

**Affiliations:** 1 Universidade de Brasília Universidade de Brasília Faculdade de Ciências da Saúde Laboratório de Farmacologia Molecular Brasília DF Brasil Laboratório de Farmacologia Molecular, Faculdade de Ciências da Saúde, Universidade de Brasília (UnB), Brasília, DF, Brasil; 2 Universidade de Brasília Universidade de Brasília Hospital Universitário de Brasília Unidade de Endocrinologia Brasília DF Brasil Unidade de Endocrinologia, Hospital Universitário de Brasília, Universidade de Brasília (UnB), Brasília, DF, Brasil

## Abstract

Pheochromocytoma (PCC) is a tumor derived from adrenomedullary chromaffin cells. Prognosis of malignant PCC is generally poor due to local recurrence or metastasis. We aim to report a case of malignant PCC with 18-year survival and discuss which factors may be related to mortality and long-term survival in malignant pheochromocytoma. The patient, a 45-year-old man, reported sustained arterial hypertension with paroxysmal episodes of tachycardia, associated with head and neck burning sensation, and hand and foot tremors. Diagnosis of PCC was established biochemically and a tumor with infiltration of renal parenchyma was resected. No genetic mutation or copy number variations were identified in *SDHB, SDHD, SDHC, MAX* and *VHL*. Over 18 years, tumor progression was managed with ^131^I-MIBG (iodine-metaiodobenzylguanidine) and ^177^Lutetium-octreotate therapy. Currently, the patient is asymptomatic and presents sustained stable disease, despite the presence of lung, para-aortic lymph nodes and femoral metastases. Adequate response to treatment with control of tumor progression, absence of significant cardiovascular events and other neoplasms, and lack of mutations in the main predisposing genes reported so far may be factors possibly associated with the prolonged survival in this case. Early diagnosis and life-long follow-up in patients with malignant pheochromocytoma are known to be crucial in improving survival.

## INTRODUCTION

Pheochromocytoma (PCC) is a tumor derived from adrenomedullary chromaffin cells, which produce catecholamines, such as norepinephrine, epinephrine and dopamine. Approximately 15-20% of chromaffin-cell tumors arise from extra-adrenal chromaffin cells, and they are called paragangliomas (1). PCCs are rare, with an incidence of 2 to 8 per 1 million adults, and correspond to 5% of all adrenal incidentalomas (2).

The clinical relevance of these tumors is based on their potential of secreting cathecolamines, causing cardiovascular morbidity and mortality. Around 0.2% to 1% of patients with hypertension have chromaffin-cell tumors. These tumors may also extend into adjacent organs, causing mass-effect symptoms (1,2).

Malignant pheochromocytomas are defined as those with metastases, evidence of local invasion, or recurrence. The main sites of metastasis are lymph nodes (80% of patients), bones (50-70%), liver (50%), lungs (30-50%), and kidney. Approximately 10% to 25% of PCCs are malignant (2–4).

There is no available method to accurately predict the malignant potential of these tumors when they present without metastases. Tumors larger than 5 cm, location of the primary lesion, local invasion, tumor necrosis, high cellularity, nuclear pleomorphism and hyperchromasia are some characteristics that may suggest malignancy, but distant metastases remain the only widely accepted malignancy criterion (3,5). The pattern of cathecolamine secretion is also not useful to differentiate benign from malignant pheochromocytomas, despite previous reports claiming that dopamine levels may be higher in malignant cases (6).

The prognosis of malignant PCC is generally poor due to local recurrence or metastasis, with 5-year survival rates varying between 40% e 70%, and median survival of 7 years (7,8). Nevertheless, there are few reports of patients with malignant PCC surviving for more than 15 years after diagnosis (6,8–24). We report a case of malignant PCC with 18-year survival and discuss which factors may be related to mortality and long-term survival in malignant pheochromocytoma.

## CASE REPORT

The patient, a 45-year-old man, reported sustained arterial hypertension with paroxysmal episodes of tachycardia, associated with burning sensations in the head and neck, and hand and foot tremors. On the first consultation, blood pressure was 220 x 160 mmHg, the heart rate was 58 bpm and a systolic murmur 2+/6+ in mitral area was noticed, radiating to the left sternal border. No relevant family history was reported. His father died of prostate cancer at age 65, but there were no other cases of cancer in the family.

An abdominal computed tomography (CT) scan showed compression of the left kidney by a tumor measuring 14.8 x 9.8 cm. At that time (1998), the concentrations of total catecholamines and vanillylmandelic acid in 24-hour urine sample were 1875 μg/24h (normal range: < 100 μg/24h) and 86.8 mg/24h (normal range: < 8 mg/24h), respectively. Adrenalectomy and nephrectomy were performed, accompanied by splenectomy and resection of the tail of the pancreas due to tumor infiltration. Histopathological examination was consistent with pheochromocytoma with vascular invasion and infiltration of renal parenchyma.

After two years, the patient presented negative biochemical evaluation for pheochromocytoma, and the only reported symptom was intestinal subocclusion, which was clinically treated. However, an abdominal CT scan demonstrated recurrence of a tumor measuring 8 x 6 cm, in the left renal bed, which was resected. In subsequent years, ^131^I-MIBG (iodine-metaiodobenzylguanidine) scintigraphy and abdominal and pelvic CT scans showed multiple enlarged para-aortic lymph nodes, compatible with metastasis, as well as metastatic nodules in the left adrenal bed and left lung base. Over twelve years, treatment was performed with four therapeutic doses of ^131^I-MIBG (two, seven, eleven and thirteen years after the second surgery), totaling a cumulative dose of 700 mCi (first dose of 100 mCi and the others of 200 mCi).

**Figure 1:**
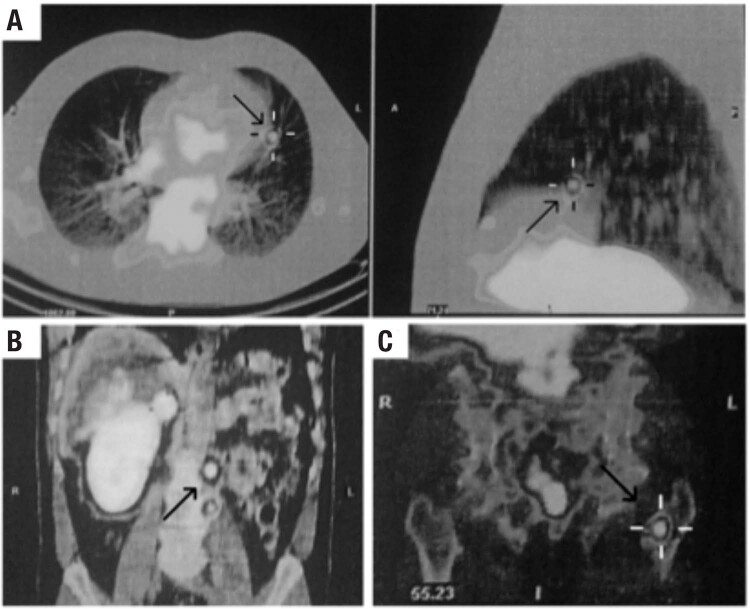
^177^Lutetium-Octreotate Single-Photon Emission Computed Tomography (SPECT) revealing areas of radiopharmaceutical hyperconcentration (shown by black arrows) in the lungs (**A**, axial and sagittal view), left para-aortic lymph nodes (**B**, coronal view), and left femoral intertrochanteric region (**C**, coronal view).

In the last seven years, however, persistently elevated levels of urinary normetanephrines have been noted. The last magnetic resonance imaging (MRI) revealed the presence of node formations on the left para-aortic region and on the topography of the ipsilateral adrenal gland. Recently, ^111^Indium-octreotide scintigraphy was performed, followed by ^177^Lutetium-octreotate therapy, and the last whole body scan after the second therapeutic dose (200 mCi) of ^177^Lutetium-octreotate demonstrated the presence of neuroendocrine lesions in the lungs, left para-aortic lymph nodes and in the intertrochanteric region of the left femur, suggesting active disease (Figure 1). The patient, currently 63 years old, remains asymptomatic and normotensive, under use of antihypertensive medication (Enalapril 40 mg/day).

In face of the malignant presentation, molecular studies were conducted to screen mutations in five genes related to pheochromocytoma: *SDHB, SDHD, SDHC, MAX* and *VHL*. Genomic DNA was extracted from 12 mL of EDTA-anticoagulated peripheral blood, by the salting out method. All coding exons and flanking intronic regions of *SDHB, SDHD, SDHC, MAX* and *VHL* were amplified and automatically sequenced. Since no point mutations were found by the Sanger sequencing, analysis for large deletions in *SDH* and *VHL* was included. Multiplex Ligand-Probe Amplification (MLPA) was performed using commercially available kits, P226 for *SDH*, and P016 for *VHL* (MRC Holland^®^). No mutation or copy number variations were identified in the assessed genes.

## DISCUSSION

We report a rare case of malignant pheochromocytoma with 18 years of follow-up, during which the patient has been asymptomatic and has referred a good quality of life after treatment with a cumulative dose of 700 mCi of ^131^I-MIBG, followed by ^177^Lutetium-octreotate therapy. While the diagnosis of a malignant pheochromocytoma was suspected in 1998 when an invasive 14.8-cm adrenal tumor was resected, the confirmation of malignancy came with early postoperative evidence of locoregional recurrence followed by multiple para-aortic lymph nodes metastasis. Despite the anatomical evidence of relatively stable metastatic disease, no evidence of cathecolamine hypersecretion was noted for approximately 10 years. Moreover, even though urinary normetanephrine levels have slowly increased over the last 7 years, the patient remains clinically controlled.

According to recent guidelines, the initial diagnosis of pheochromocytoma is based on the assessment of increased cathecolamines or metabolites (metanephrines) in a plasma sample or in a 24h urine collection, followed by localization of the tumor using imaging techniques, such as CT, T2-weighted MRI and I-MIBG scintigraphy (3). In the present case, MRI and I-MIBG scintigraphy have shown complementary usefulness in the clinical follow up and identification of disease recurrence, as previously reported (25).

Genetic testing is recommended in all PCC patients, since it may guide genetic counselling and ensure a personalized approach to patient management. Although approximately 14 confirmed genes are associated with pheochromocytoma and paraganglioma susceptibility, the genes investigated in the present case – *SDHB, SDHC, SDHD*, *VHL* and *MAX* – have been the main reported genes associated with malignant PCC reported so far, according to the Endocrine Society Clinical Practice Guideline (1). Metastatic disease is one of the features that indicates likelihood of a hereditary cause, and it is recommended that patients with malignant PCC should undergo genetic testing for *SDHB, SDHC, SDHD*, *VHL* and *MAX* (1,26). In the presented case, we performed genetic screening of those main malignancy predisposing genes, but no germline mutations or copy number variations were identified. Accordingly, it has been shown that only 40% of patients carry germline mutations in the susceptibility genes, and up to two thirds of the remaining sporadic PCCs have somatic mutations (26).

The prevalence of germline mutations in *SDHB, SDHC, SDHD*, *VHL* and *MAX* in PCC and paraganglioma patients is, respectively, 10%, 1%, 9%, 7%, and 1%. *SDHx* mutations are associated with inappropriate activation of hypoxic signaling leading to angiogenesis, metabolic alterations and tumorigenesis. *SDHB* mutations are especially related to sporadic malignant PCC with poor prognosis, and up to 40% of patients with metastatic disease harbor mutations in this gene. Hypoxic signaling is also disrupted by loss-of-function mutations in *VHL*, which cause von Hippel-Lindau disease, a syndrome characterized by tumors in several organs, including kidney, central nervous system, pancreas and adrenal gland. *MAX* mutations promote upregulation of the MYC signaling, a key oncogenic pathway, leading to familial pheochromocytomas (1,26).

The absence of germline mutations in the main predisposing genes suggests that other mechanisms are related to the development of malignancy, particularly in indolent cases, which still has no biological markers or defined pathophysiological basis. The absence of mutations in the reported case, especially *SDHB*, may also be related to long-term survival, since *SDHB* mutations are independently related to shorter survival in patients with malignant pheochromocytoma (26). Intriguingly, there are few reports of malignant pheochromocytoma patients harboring *SDHB* mutations with prolonged survival (17,22). Whether the absence of germline mutations in the genes previously associated with a malignant behavior represents a protective factor against aggressive disease must be further investigated.

Curative treatment for malignant PCC is not available yet, and surgery is focused on reducing hormonal activity and palliating local complications. MIBG-radiotherapy, radiolabeled somatostatin analogues (e.g. octreotide), and chemotherapy are other available therapeutic options. Conventional chemotherapeutic regimens (e.g. cyclophosphamide, vincristine, and dacarbazine) provide limited efficacy (27).

Several studies have investigated the use of radiolabeled MIBG for treatment of malignant PCC. Dose regimen and tumor response are variable, as there is no established agreed-upon protocol. A phase II study involving 50 patients with malignant pheochromocytoma or paraganglioma treated with high-dose ^131^I-MIBG showed overall 5-year survival rate of 64% and sustained stable disease in 8% of patients (28). Another study reported median survival of 4.7 years for patients with malignant PCC after treatment with high-dose ^131^I-MIBG (29).

In the present case, the patient responded to treatment with ^131^I-MIBG for 12 years and, more recently, to ^177^Lutetium-octreotate therapy. Currently, the patient presents sustained stable disease, despite the presence of metastases in the lung, para-aortic lymph nodes and, more recently, femoral metastasis. Repeated intermediate-dosage of ^131^I-MIBG is considered an efficient and well-tolerated treatment in patients with malignant PCC, but the role of ^131^I-MIBG in longevity remains to be elucidated in patients with long-term survival (19).

The most frequent cause of death in malignant pheochromocytoma is tumor progression, highlighting the importance of controlling tumor growth in disease management. Cardiovascular manifestations related to catecholamine hypersecretion account for 30% of mortality (4). The main cardiovascular events associated with mortality are myocardial infarction, heart failure, cerebrovascular complications, and hypertensive or hypotensive crisis associated with operations for unrelated conditions (7).

Other cancers are also associated with mortality in PCC. These additional neoplasms may occur in the context of genetic syndromes, such as von Hippel-Lindau syndrome and neurofibromatosis type 1. They also may be sporadic, considering that presence of pheochromocytoma is associated with increased risk of developing other malignancies (melanoma, carcinoma of uterine cervix, liver/biliary tract and central nervous system tumors) (30). So far, no additional or secondary neoplasm has been identified in our case. Control of tumor progression, as well as the absence of significant cardiovascular events and other cancers may have contributed to the prolonged survival in the reported case.

An extensive literature search from 1970 to 2016 revealed 42 cases of malignant pheochromocytomas and paragangliomas with more than 15 years of survival (Table 1). It is noteworthy that a significant number of malignant cases with prolonged survival (11 of 42; 26%) were described more than 25 years ago, when imaging and radionuclide-based therapies were not widely available in clinical practice. Overall, among 31 cases of malignant PCCs with prolonged survival in which the primary tumor localization was provided, the majority were unilateral adrenal tumors (62.5%; n = 20), followed by extra-adrenal (35.5%; n = 11), and bilateral tumors (3.2%; n = 1). Only two studies (17,22) performed genetic analysis, and out of four cases in which genetic screening was performed in apparently sporadic malignant PCC, three presented *SDHB* mutations, and one patient was negative for *SDHB*, *SDHD*, *RET* and *VHL* mutations. The variability in clinical presentation and rarity of cases with prolonged survival impair the establishment of definitive factors associated with better outcomes and long-term survival in malignant PCC.

**Table 1:** Reports of malignant pheochromocytomas and paragangliomas with more than 15 years of survival

No.	Age at diagnosis (years)	Location of primary tumor	Tumor size (cm)	Site of metastasis	Genetic testing	Survival time (years)	Reference
1	43	Adrenal Uni	NA	Pleura	NA	20	Traub and Rosenfeld (1970) (9)
2	NA	NA	NA	NA	NA	17	
3	NA	NA	NA	NA	NA	20	Remine and cols. (1975) (10)
4	NA	NA	NA	NA	NA	20	
5	NA	NA	NA	NA	NA	21	
6	NA	NA	NA	Lung	NA	25	Van den Broek and De Graeff (1978) (11)
7	7	Adrenal Uni	NA	Left renal hilus	NA	21	Abemayor and cols. (1980) (12)
8	NA	NA	NA	NA	NA	>20	Brennan and Kaiser (1982) (13)
9	NA	NA	NA	NA	NA	23	
10	NA	NA	NA	Liver	NA	17	Okada and cols. (1990) (14)
11	NA	NA	NA	NA	NA	22	Mornex and cols. (1992) (15)
12	44	Adrenal Uni	NA	Lumbar vertebrae and ilium	NA	26	Yoshida and cols. (2001) (16)
13	32	Extra-adrenal	NA	Liver and vena cava	*SDHB*	30	Young and cols. (2002) (17)
14	NA	NA	NA	Bone	NA	30	Arias Martinez and cols. (2003) (24)
15	19	Adrenal Bi	NA	Liver	NA	29	Mózes and cols. (2003) (18)
16	43	Adrenal Uni	9	Liver and retroperitoneum	NA	16	Lam and cols. (2005) (19)
17	18	Extra-adrenal	NA	Bone	NA	21	
18	30	Adrenal Uni	NA	Lung	NA	34	
19	17	Extra-adrenal	NA	Regional	NA	25	
20	25	Adrenal Uni	NA	Liver	NA	17	
21	39	Adrenal Uni	NA	Liver	NA	17	
22	42	Adrenal Uni	NA	Regional	NA	18	Sisson and cols. (2006) (20)
23	23	Adrenal Uni	NA	Liver	NA	36	
24	10	Extra-adrenal	NA	Bone	NA	32	
25	36	Adrenal Uni	NA	Lung	NA	17	
26	69	Extra-adrenal	NA	Liver	NA	16	
27	15	Adrenal Uni	NA	Bone	NA	45	
28	51	Adrenal Uni	11x12x11	Lung and bone	NA	20	Huang and cols. (2007) (6)
29	34	Adrenal Uni	22	NA	NA	18	Szalat and cols. (2011) (21)
30	49	Adrenal Uni	NA	Lung	NA	19	
31	47	Adrenal Uni	3.2	Kidney and retroperitoneum	NA	18	Choi and cols. (2015) (8)
32	26	Adrenal Uni	NA	Lung	NA	18	
33	14	Adrenal Uni	NA	Liver, lung, duodenum, bone	NA	25	
34	47	Extra-adrenal	NA	NA	NA	17	
35	50	Extra-adrenal	NA	NA	NA	25	
36	15	Extra-adrenal	NA	NA	NA	21	
37	26	Adrenal Uni	NA	NA	NA	31	Rutherford and cols. (2015) (22)
38	56	Adrenal Uni	NA	NA	*SDHB*	16	
39	13	Extra-adrenal	NA	NA	*SDHB*	25	
40	31	Adrenal Uni	NA	NA	Negative	18	
41	32	Extra-adrenal	NA	NA	NA	26	
42	53	Extra-adrenal	NA	Anterior cranial fossa and cervical lymph nodes	NA	22	Belgioia and cols. (2016) (23)

Uni: unilateral; Bi: bilateral; NA: not available.

This case report and literature review carefully illustrate that regular clinical monitoring with imaging and biochemical evaluation of malignant pheochromocytomas is essential to address tumor behavior, and to ensure prompt intervention when complications or tumor recurrence arise. In addition, early diagnosis and life-long follow-up are crucial for improving survival. Adequate response to treatment with control of tumor progression, absence of significant cardiovascular events and other neoplasms, and lack of mutations in the main predisposing genes reported so far might be associated with the prolonged survival in the presented case. Further studies involving a larger number of cases are needed to compare the clinical, biochemical and molecular signatures of malignant pheochromocytomas with indolent *versus* aggressive behavior and treatment response.
